# The mitochondrial genome of a minute springtail species *Megalothorax incertus* (Collembola: Neelipleona: Neelidae)

**DOI:** 10.1080/23802359.2021.1955637

**Published:** 2021-07-19

**Authors:** Yao Ma, Cheng-Wang Huang, Yun-Xia Luan, Wan-Jun Chen

**Affiliations:** a Department of Biotechnology, College of Life Science and Technology, Huazhong University of Science and Technology, Wuhan, China; b CAS Key Laboratory of Insect Developmental and Evolutionary Biology, CAS Center for Excellence in Molecular Plant Sciences, Chinese Academy of Sciences (CAS), Shanghai, China; c Guangdong Provincial Key Laboratory of Insect Development Biology and Applied Technology, Institute of Insect Science and Technology, School of Life Sciences, South China Normal University, Guangzhou, China

**Keywords:** Neelipleona, mitochondrial genome, parthenogenesis, *Megalothorax incertus*

## Abstract

In this study, the complete mitochondrial genome for the *Wolbachia* infected parthenogenetic collembola *Megalothorax incertus* Börner, 1903 was determined. It represents the first report of a complete mitochondrial genome from Neelipleona, one of the four orders of Collembola. The circularized 14,994 bp mitochondrial genome sequence consists of canonical 37 mito-genes, including 13 protein-coding genes (PCGs), 22 tRNA genes, and two rRNA genes. The base composition of the majority strand (same direction as most of the mitochondrial genes) is 32.0% for A, 24.1% for C, 11.9% for G, and 32.1% for T. The phylogenetic trees inferred from 13 PCGs using the Maximum-likelihood (ML) and Bayesian inference (BI) methods suggested that Neelidae is basal to the remaining springtails. This mitochondrial genome provides new insights to decipher the phylogeny of Collembola.

Neelipleona is an understudied group of Collembola. It has the fewest described species (about 60 species according to http://www.collembola.org, last accessed 2021 Jun 02) among four present recognized Collembola orders (Deharveng 2004). Neelipleona has a single family, Neelidae, which plays a significant role in revealing collembolan’s evolutionary history. The molecular sequences from Neelidae species are relatively scarce, which impedes solving its phylogenetic status. For example, the close affinity between Neelipleona and Symphypleona was traditionally supported by morphological characters like globular shape, neosminthuroid setae, mucro gutter-like etc. (Schneider et al. 2011), which needs to be further tested from the molecular phylogenetic perspective (Xiong et al. 2008). What’s more, *Wolbachia* infection was detected from *Megalothorax incertus* Börner, 1903 in our previous studies (Ma et al. 2017). As both *Wolbachia* and mitochondrial genome are maternally inherited, whether coevolution of them contributed to the parthenogenetic lifestyle remains to be investigated. Here we determined the mitochondrial genome of *M. incertus* using a combination of multiple experimental and bioinformatic procedures, including long-range PCR, whole genome amplification, high-throughput shotgun sequencing and assembly. The annotated mitochondrial genome sequence of *M. incertus* was deposited in NCBI database with the accession of MW916537.

Specimens of *M. incertus* were first collected from Shanghai Expo Houtan Park, Shanghai, China (31°18'N, 121°47'E) by Yan Gao and colleagues in 2012. It was then cultured in the laboratory and confirmed as parthenogenetic through direct observation of reproduction from single unfertilized females. A specimen was deposited at Shanghai Entomological Museum, Chinese Academy of Sciences, Shanghai, China (http://www.shem.com.cn/, contact person: Cheng-Wang Huang, cwhuang@cemps.ac.cn) under the voucher number YIN20210611. Total DNA was extracted for four individuals (labeled as D1–D4) separately with the DNeasy Blood and Tissue Kits (Qiagen, Germany). The barcoding region of *cox1* gene was PCR-amplified using universal primer HCO/LCO and sequenced by sanger sequencing to verify the species identity (Simon et al. 1994). Among the four DNA samples, one was used for whole genome amplification (WGA) (sample D1) and another for long-PCR amplification (sample D3), which were followed by illumina shotgun sequencing. The WGA were performed using the REPLI-g Cell WGA & WTA Kit (Qiagen, Germany) following the manufacture’s recommendations. The amplified genomic DNA was subjected to library preparation and sequencing on illumina HiSeq X Ten System, being carried out at Sangon Biotech (Shanghai) Co., Ltd as a commercial service. The circularized mitochondrial genome sequence was assembled from over 7 Gb of 150 bp pair-end reads using Novoplasty (version 3.6) (Dierckxsens et al. 2016), with *cox1* sequence as seed, and further annotated using MITOS2 web portal (Donath et al. 2019). The annotations were also manually checked by comparing with the reported collembolan mitochondrial genomes. The Long PCR products of the region from *cox1* to *cytb* (∼9Kb, covering 11 of 13 mitochondrial PCGs) in four overlapped fragments were also achieved using conserved mitochondrial universal primers (Simon et al. 1994). The raw sequencing reads of those mixed PCR products were assembled with MEGAHIT software (Li et al. 2015). The assembled single 9Kb contig verified the sequence accuracy of Novoplasty assembled mitochondrial genome of the same region.

The mitochondrial genome of *M. incertus* is 14,994 bp in length, like typical hexapod mitogenomes, displaying a general A + T-bias (A + T content: 64.0%). The AT-skew and GC-skew were calculated as AT-skew = (A − T)/(A + T) and GC-skew = (G − C)/(G + C) to assess the strand asymmetry. The AT-skew is −0.001 and GC-skew is −0.341, which suggests A and T has a similar base content while C is favored compared to G. The mitochondrial genome of *M. incertus* encodes 37 typical mitochondrial genes common to Metazoa. However, the *trnR* gene was not predicted by MITOS2 initially. After comparing the sequence in the region between *trnA* and *trnN* with other collembolan *trnR* sequences, a potential abbreviated *trnR* were predicted in this locus, the *trnR* identity was also supported by harboring a presumed ‘UCG’ anticodon in the RNAfold predicted secondary structure (Lorenz et al. 2011). Four PCGs (cox*1*, *cox2*, *atp6,* and *nad5*) use incomplete stop codons (TA-/T–), most likely post-transcriptionally restored into complete stop codons. Eleven out of 13 PCGs were annotated to use canonical ATN start codons (five using ATT, four using ATG, and two using ATA). Unexpectedly, *cox1* and *nad5* might use TTG as the start codon. The gene order is the same as the ancestral gene order for Pancrustracea and in agreement with another nearly complete mitochondrial genome from *Neelus murinus* Folsom, 1896 (Neelipleona) (MH155200) (Leo et al. 2019).

All available complete mitochondrial genomes of Collembola were retrieved from the GenBank RefSeq database in March 2021. The nearly complete mitochondrial genome of *N. murinus* was also added to the phylogenetic analysis. The *Japyx solifugus* (Diplura, Japygidae) was used as an outgroup. Please refer to Figure 1 for accession numbers of each sequence. The 13 PCGs were extracted from GenBank format files using an in-house Python script. The amino acid sequences were aligned individually with MUSCLE (Edgar 2004). Nucleotide sequences were then retro-aligned using the PAL2NAL script (Suyama et al. 2006). Three datasets (nt12, nt123, aa) were used for phylogenetic analysis. All data sets were analyzed using both RAxML and MrBayes algorithms embedded in GENEIOUS R11 software using default settings. All analyses yield almost the same topology, represented by the nt12 RAxML tree shown in Figure 1. The *M. incertus* clustered with *N. murinus* and in support of monophyly of Neelipleona. Neelipleona is also recovered in a basal position in Collembola. The sister relationship between Neelipleona and Symphypleona was not supported.

**Figure 1.**
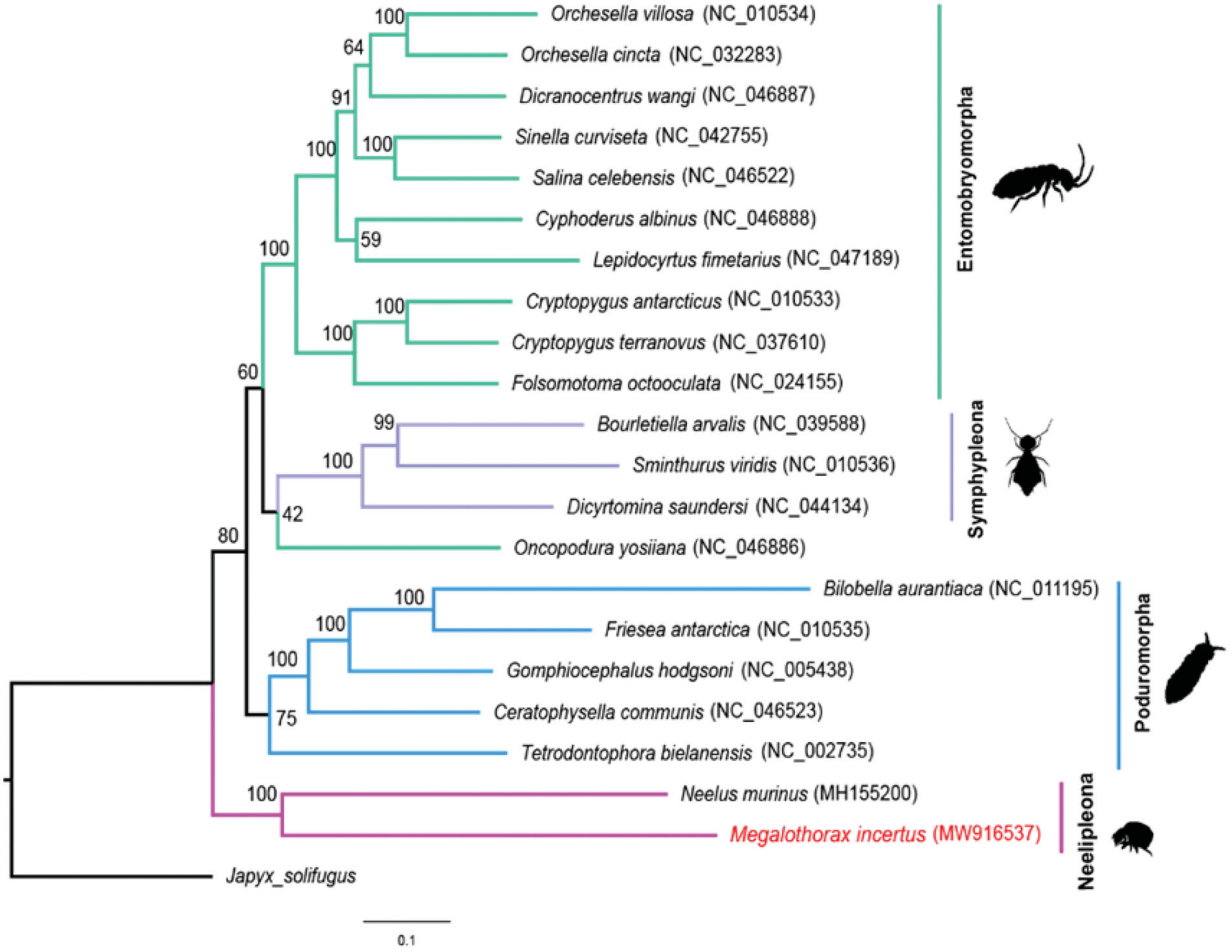
Maximum likelihood estimated tree using RAxML with nucleotide sequences of 13 PCGs. Only the 1st and 2nd codon positions were used in the tree, while all codon positions also result in the same topology. The accession numbers were given in the parentheses following each species names. The number on each branch represents the bootstrap support for this node.

## Data Availability

The genome sequence data that support the findings of this study are openly available in GenBank of NCBI at (https://www.ncbi.nlm.nih.gov/) under the accession no. MW916537. The associated BioProject, SRA, and Bio-Sample numbers are PRJNA738273, SRR14826175, and SAMN19717180 respectively.

## References

[CIT0001] Deharveng L. 2004. Recent advances in Collembola systematics. Pedobiologia. 48(5–6):415–433.

[CIT0002] Dierckxsens N, Mardulyn P, Smits G. 2016. NOVOPlasty: de novo assembly of organelle genomes from whole genome data. Nucleic Acids Res. 45(4):e18. 10.1093/nar/gkw955 PMC5389512 28204566

[CIT0003] Donath A, Jühling F, Al-Arab M, Bernhart SH, Reinhardt F, Stadler PF, Middendorf M, Bernt M. 2019. Improved annotation of protein-coding genes boundaries in metazoan mitochondrial genomes. Nucleic Acids Res. 47(20):10543–10552. 31584075 10.1093/nar/gkz833 PMC6847864

[CIT0004] Edgar RC. 2004. MUSCLE: multiple sequence alignment with high accuracy and high throughput. Nucleic Acids Res. 32(5):1792–1797. 15034147 10.1093/nar/gkh340 PMC390337

[CIT0005] Leo C, Carapelli A, Cicconardi F, Frati F, Nardi F. 2019. Mitochondrial genome diversity in Collembola: phylogeny, dating and gene order. Diversity. 11(9):169.

[CIT0006] Li D, Liu C-M, Luo R, Sadakane K, Lam T-W. 2015. MEGAHIT: an ultra-fast single-node solution for large and complex metagenomics assembly via succinct de Bruijn graph. Bioinformatics. 31(10):1674–1676. 25609793 10.1093/bioinformatics/btv033

[CIT0007] Lorenz R, Bernhart SH, Höner Zu Siederdissen C, Tafer H, Flamm C, Stadler PF, Hofacker IL. 2011. ViennaRNA package 2.0. Algorithms Mol Biol. 6(1):26. 22115189 10.1186/1748-7188-6-26 PMC3319429

[CIT0008] Ma Y, Chen WJ, Li ZH, Zhang F, Gao Y, Luan YX. 2017. Revisiting the phylogeny of Wolbachia in Collembola. Ecol Evol. 7(7):2009–2017. 28405268 10.1002/ece3.2738 PMC5383468

[CIT0009] Schneider C, Cruaud C, D’Haese CA. 2011. Unexpected diversity in Neelipleona revealed by molecular phylogeny approach (Hexapoda, Collembola). Soil Organisms. 83(3):383–398.

[CIT0010] Simon C, Frati F, Beckenbach A, Crespi B, Liu H, Flook P. 1994. Evolution, weighting, and phylogenetic utility of mitochondrial gene sequences and a compilation of conserved polymerase chain reaction primers. Ann Entomol Soc Am. 87(6):651–701.

[CIT0011] Suyama M, Torrents D, Bork P. 2006. PAL2NAL: robust conversion of protein sequence alignments into the corresponding codon alignments. Nucleic Acids Res. 34(Web Server issue):W609–W12. 16845082 10.1093/nar/gkl315 PMC1538804

[CIT0012] Xiong Y, Gao Y, Yin W-y, Luan Y-x. 2008. Molecular phylogeny of Collembola inferred from ribosomal RNA genes. Mol Phylogenet Evol. 49(3):728–735. 18835455 10.1016/j.ympev.2008.09.007

